# Characteristics of Long-COVID care centers in Italy. A national survey of 124 clinical sites

**DOI:** 10.3389/fpubh.2022.975527

**Published:** 2022-08-19

**Authors:** Marco Floridia, Tiziana Grassi, Marina Giuliano, Dorina Tiple, Flavia Pricci, Marika Villa, Andrea Silenzi, Graziano Onder

**Affiliations:** ^1^National Center for Global Health, Istituto Superiore di Sanità, Rome, Italy; ^2^Department of Cardiovascular, Endocrine-Metabolic Diseases and Aging, Istituto Superiore di Sanità, Rome, Italy; ^3^Department of Neuroscience, Istituto Superiore di Sanità, Rome, Italy; ^4^Ministry of Health, General Directorate for Health Prevention, Rome, Italy

**Keywords:** COVID-19, Long-COVID, Post-COVID, health services, health care

## Abstract

**Background:**

Despite the growing clinical relevance of Long-COVID, there is minimal information available on the organizational response of health services to this condition.

**Methods:**

A national online survey of centers providing assistance for Long-COVID was implemented. Information collected included date of start of activity, target population, mode of assistance and of referral, type and number of specialists available, diagnostic and instrumental tests, use of telemedicine and of specific questionnaires.

**Results:**

Between February and May 2022, 124 centers completed the survey. Half of them were situated in northern Italy. Most (88.9%) provided assistance through either outpatient visits or day hospital services. Eleven (8.9%) assisted pediatric patients. Access to centers included scheduled visits for previously hospitalized patients (67.7%), referral from primary care (62.1%), from other specialists (46.8%), and, less commonly, from other services. Almost half of the centers (46.3%) started their activity early in the pandemics (March-September 2020). Almost all (93.5%) communicated with primary care physicians, and 21.8% used telemedicine tools. The mean number of patients followed was 40 per month (median 20, IQR 10-40). In most cases, the center coordinator was a specialist in respiratory diseases (30.6%), infectious diseases (28.2%), or internal medicine (25.0%). At least half of the centers had specialistic support in cardiology, respiratory diseases, radiology, infectious diseases, neurology and psychology, but roughly one quarter of centers had just only one (14.5%) or two (9.7%) specialists available. The clinical assessment was usually supported by a wide range of laboratory and instrumental diagnostics and by multidimensional evaluations.

**Conclusions:**

Most of the centers had an articulate and multidisciplinary approach to diagnosis and care of Long-COVID. However, a minority of centers provided only single or dual specialistic support. These findings may be of help in defining common standards, interventions and guidelines that can reduce gaps and heterogeneity in assistance to patients with Long-COVID.

## Introduction

As of May 2022, more than 500 million confirmed cases of SARS-CoV-2 infection have been reported globally to the WHO (1), and there is increasing evidence that in a substantial proportion of such cases, Coronavirus 19 disease (COVID-19) will lead to prolonged health consequences following acute infection. This syndrome, generally defined Long-COVID when subacute and/or chronic symptoms of SARS-CoV-2 infection extend beyond three months, is still incompletely defined in terms of clinical characteristics (2, 3). Published studies were largely heterogeneous in design, size, population studied, length of follow up and disease definition, making very difficult to estimate the real prevalence and incidence of this condition (4, 5). However, even assuming a population-based prevalence for adults around 10% (roughly the center of the large interval of prevalences reported), Long-COVID will have a substantial public health and social impact, posing new challenges to the health systems, that will have to respond providing multidisclipinary care to a large number of patients presenting with an extreme clinical heterogeneity (6–8).

Despite the relevance of the subject, there is only sporadic information on how the health systems reacted implementing new facilities and pathways of care specifically dedicated to Long-COVID (9, 10). We here report the results of a survey held in early 2022 on a national basis to define the number and characteristics of centers assisting patients with Long-COVID manifestations in Italy.

## Methods

The survey is part of the larger project “Analysis and strategies of response to the long-term effects of COVID-19 infection (Long-COVID)” funded by the National Center for Disease Prevention and Control of the Italian Ministry of Health in 2021 and started in 2022. An online dedicated platform for the registration of centers specifically dedicated to diagnosis and care of Long-COVID was implemented for the survey. The questionnaire items were defined through expert consultation, and the survey was publicized through distribution to regional health authorities, hospital networks, and scientific societies. Registered users had access to the compilation of the questionnaire through a two-step process that included provision of specific credentials and a one-time password system. Participation to the survey occurred on a voluntary basis. The questionnaire included items on date of start of activity of the center, case volume, target population (adult, pediatric, or both) modality of referral, type and number of specialists available, diagnostic tests, multidimensional evaluations, use of telemedicine and use of specific questionnaires. The survey started on February 2022 and data for the present analysis were extracted on May 13, 2022. Data are reported descriptively and summarized as proportions for categorical variables and as means and medians with interquartile ranges (percentiles 25-75) for quantitative variables. Mean values were compared by Student's T test. Analyses were performed using the SPSS software, version 27.0 (IBM Corp, 2017, Armonk, NY, US).

## Results

One hundred and thirty seven centers from 17 Italian regions and two autonomous provinces registered on the online platform. Among them, 124 (90.5%) from 16 regions and two autonomous provinces completed the online questionnaire. Their general characteristics are shown in Table 1. Half of the respondents (62/124) were from northern Italy (24 from the Lombardy region alone, representing 19.3% of all centers); 36 were from central Italy (29%), and 26 from southern Italy (21%). Most of the centers provided essentially outpatient visits or day hospital services, with only five per cent assisting patients in regimens of hospital admission. Overall, nine per cent of centers assisted pediatric patients. Access to centers used multiple pathways, that included scheduled visits for previously hospitalized patients (67.7%), referral from primary care (62.1%) or from other specialists (46.8%), and, less commonly, access from other territorial services. Almost half of the centers (46.3%) started their activity early in the pandemics (March-September 2020), but the implementation of new Long-COVID centers appeared to be a continuous process still ongoing at the time of the survey (8.9% in March-April 2022). Almost all the centers (93.5%) had communication pathways with primary care physicians, roughly 20 per cent used telemedicine tools, and four per cent provided domiciliary care. In terms of case volume, centers followed a mean of 40.0 patients per month (median 20, IQR 10-40), with 22.3 first visits per month (median 15, IQR 7-25) and 23.5 follow up visits per month (median 10, IQR 5-27).

**Table 1:** General characteristics of the Long-COVID care centers.

	***N***	**%**
**Location**		
North	62	50.0
Center	36	29.0
South	26	21.0
University Hospital	40	32.2
**Predominant patient care**		
Ambulatory visits	98	79.2
Day hospital	12	9.7
Rehabilitation	8	6.5
Inpatients (at least 1 night)	6	4.8
**Population**		
Adult	113	91.1
Pediatric	6	4.8
Both	5	4.0
**Mode of access**		
Scheduled for previously hospitalized patients	84	67.7
Referral by primary care physician/pediatrician	77	62.1
Referral by specialist	58	46.8
Other^**^	10	8.1
**Date of start of activity**		
March–September 2020	57	46.3
October 2020–February 2021	27	21.9
March–September 2021	13	10.6
October 2021–February 2022	15	12.2
March–April 2022	11	8.9
**Services provided to patients at home**		
Domiciliary care	5	4.0
Telemedicine	27	21.8
**Other support provided**		
Reports to primary care physician/pediatrician	116	93.5
Information on self-management to patients	116	93.5
Contacts for support by self-help groups	17	13.7

** Territorial services of prevention and care, emergency departments, direct access of patients.

**Figure 1:**
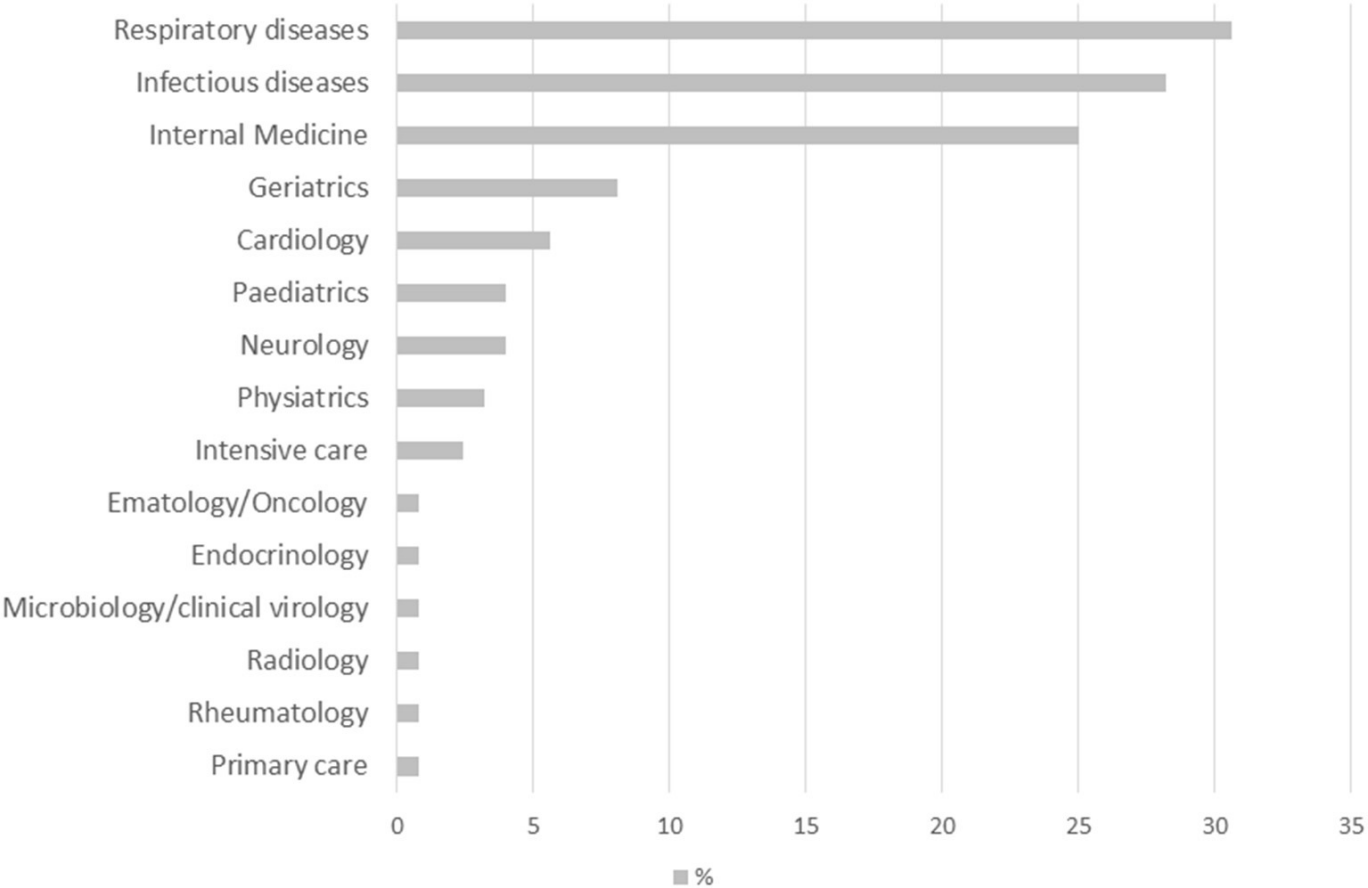
Specialty of the clinical coordinator/head of the center.

The specialty of the center coordinator is shown in Figure 1. In most of the cases, the Long-COVID center coordinator was a specialist in respiratory diseases (30.6%), infectious disease (28.2%), or internal medicine (25.0%), and less commonly a geriatrician (8.1%), a cardiologist (5.6%), a pediatrician (4.0%) or a neurologist (4.0%).

The number of competences available at Long-COVID centers was highly heterogenous (median 6, IQR 3–11, range 1–15). Eighteen centers (14.5%) had only one specialist available, that was represented by an infectiologist in six cases, a pulmonologist in five, a cardiologist or a neurologist in three and by a physiatrist in one case. Thirty centers (24.2%) had only two specialists available, in most of the centers represented by pulmonologists (12), cardiologists (9) and infectiologists (8), and less commonly by neurologists (4), geriatricians (3) or other specialties.

**Figure 2:**
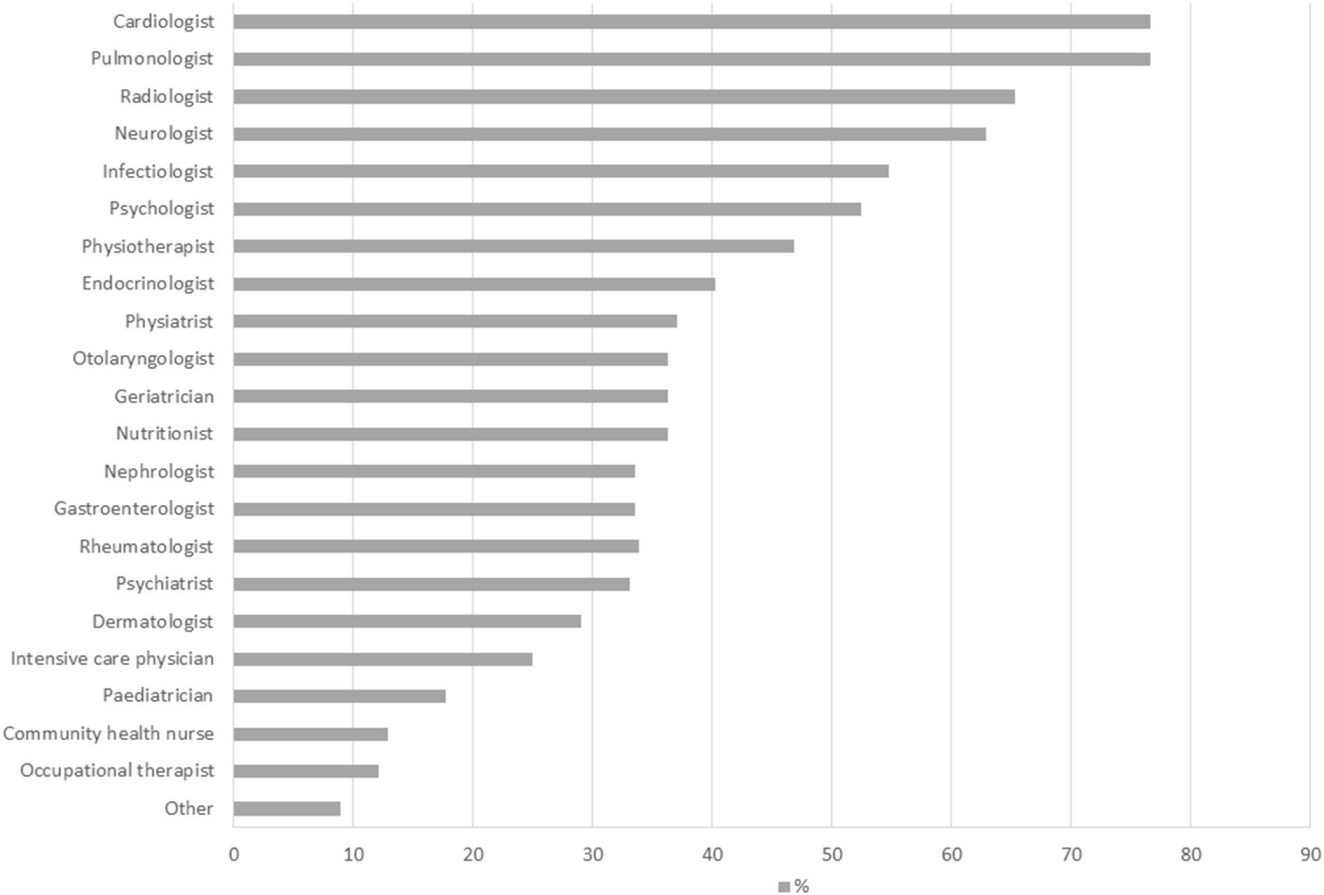
Specialist support available.

Most of the centers had multiple figures locally available. Their distribution is shown in Figure 2. At least half of the centers had specialist support in cardiology, respiratory diseases, radiology, infectious disease, neurology and psychology.

The case volume in terms of number of patients followed, first visits per month, and follow up visits per month did not differ significantly between centers with single or dual specialistic support and centers with multiple specialties available (mean number of patients followed: 45.0 vs. 38.4, respectively, *p* = 0.615; first visits per month 18.9 vs. 23.4, *p* = 0.418; follow up visits per month: 27.8 vs. 22.2, *p* = 0.552). No significant differences were also found in the mean number of patients followed between centers that started their activity early (March-September 2020) or later (October 2020-April 2022) in the pandemics, (44.8 vs. 34.3, respectively, *p* = 0.355).

The clinical diagnosis was supported by a wide range of tests and evaluations. Most of the centers provided blood testing (76.6%), six-minute walking test (74.2%), high resolution computed tomography (73.4%), electrocardiography (72.6%), heart ultrasonography (71.0%), arterial blood gas testing (71.0%) and spirometry with DLCO (66.9%). Such diagnostics were complemented by a multidimensional assessment (54.8%), by psychological evaluations (46.8%), and by assessments of functional status (46.8%), quality of life (41.9%), cognition (37.9%), and nutritional status (30.6%). The specific instruments and questionnaires used were highly variable, with interview and Hamilton scales most commonly used for psychological evaluations, Barthel, IADL/ADL or SPPB for functional status, MMSE or MoCA for cognitive assessment, EuroQol/EQ-5D or SF-36 for quality of life, and MUST/MNA for nutritional assessment.

## Discussion

We defined in this national-based survey the distribution and the characteristics of the clinical centers assisting patients with Long-COVID in Italy as of April 2022. The geographical distribution of the centers was polarized, with almost half of the centers situated in northern Italy. This area sustained the most severe burden in terms of morbidity and mortality during the first wave of the COVID epidemics. Information on the geographical distribution of Long-COVID in Italy is lacking, and we cannot define whether this distribution actually reflects a higher territorial prevalence of Long-COVID in the area most severely stressed by the first wave of the pandemic or only a different structural response of health services (e.g., for systematic follow up of patients previously hospitalized for COVID). In any case, our findings indicate a prompt organizational response, because almost half of the total centers were established during the first wave (March-September 2020). The process, however, did not stop after this phase, because new Long-COVID centers were still being implemented at the time of the survey (March-April 2022), suggesting continuous and ongoing demand for dedicated assistance.

In the vast majority of the cases, assistance was provided through outpatient visits in ambulatory structures or through day-hospital services. This suggests for most of the centers the selection of the one-stop assistance model, with visits and diagnostic procedures concentrated in a single day whenever possible. Specialized structures providing exclusively rehabilitation services were also present, although less frequently; assistance provided through hospitalization for more than one day was conversely rare. Almost ten per cent of centers assisted pediatric patients, either exclusively or in parallel with services dedicated to adults, confirming the relevance of the condition and the provision of assistance also to patients in young age. Almost all the centers had well-defined pathways of communication with primary care physicians, suggesting good connections between different level of assistance and care. Home support services were however not frequent, with only a minority of the centers using telemedicine (20%), and very few (4%) providing domiciliary care.

Most of the centers had a multidisciplinary team, with a wide range of specialists involved, most commonly cardiologists, specialists in respiratory or infectious diseases, and neurologists, accompanied by a wide range of other medical specialists, psychologists, physiotherapists, specialized nurses and occupational therapists. The competences of coordinators were more homogeneous, and mostly restricted to respiratory diseases, infectious diseases, or internal medicine. Some centers reported specifically the presence of a dedicated case manager. Although current clinical guidelines recommend a multidisciplinary approach (11, 12), there is no evidence on what specialties are essential to treat Long-COVID syndromes and Post-COVID conditions. As part of the larger project encompassing this survey, a national panel of experts will produce guidelines on management of patients and organization of services, that should be available in the next few months. The best organizational structure of the Long-COVID care centers and the optimal provisional pathways of care are still undefined, and evidence-based recommendations are lacking. Important efforts are being conducted in order to define gold standards of care and best clinical practices. Initiatives based on a multidisciplinary approach that involves specialized clinics, generalist care and patient contribution are ongoing (13). Key elements are represented by establishment of clinical networks for the collection of data, common definition of outcomes, and use of validated instruments. Regional and national public health institutions will have to ensure equal access to services and homogenous provision of care.

Roughly one quarter of the centers provided assistance through only one or two specialties. This suggests that although most of the structures were established and designed for a multidisciplinary approach, in a few cases the assistance was apparently focused on the diagnosis and care of specific manifestations of Long-COVID. An alternative explanation for the presence of single-specialty structures was the reconversion of ambulatories for infectious and respiratory diseases to services specifically dedicated to Long-COVID.

The analysis of case volume showed that each center followed a mean of 40 patients per month, with a relatively large variability. This number appeared to be relatively low. Expertise on care of Long-COVID is however still lacking, and the situation captured may represent, at least in some cases, an emergency response to a new demand of care. We think that the situation is rapidly evolving and that the number of centers, the number of patients followed at each center and the local expertise will increase with time. We cannot make inferences on the overall prevalence of Long-COVID based on these numbers, or on its possible differences by geographical area. Specifically designed studies are needed to assess prevalence of Long-COVID.

The laboratory, radiologic and instrumental support to the clinical assessment was consistent. A wide range of laboratory analyses, functional tests, instrumental evaluations, and imaging techniques, together with specific questionnaires and multidimensional assessments, were used. In particular, more than seventy per cent of centers could support the clinical assessment with the results of high-resolution computed tomography, electrocardiography, heart ultrasonography, and arterial blood gas testing; two thirds could perform spirometry with DLCO, and roughly half complemented the diagnostic process with multidimensional assessments, psychological evaluations, and assessments of functional status and quality of life. Overall, this indicates in the majority of the centers an articulate diagnostic capability able to capture the wide clinical expression of the syndrome. Improvement is however still necessary and desirable for a more comprehensive assessment. Particular attention should be given to multidimensional evaluations, whose relevance has been highlighted by several guidelines (11, 12, 14).

In terms of study limitations, the study coverage, despite the large number of respondents and the wide publicization of the survey to regional health authorities and hospital networks, remains necessarily incomplete, and not all the centers caring for Long-COVID in Italy were included. Before that survey, there was no information on centers assisting Long-COVID patients in Italy. We had no list of centers, and the survey was publicized contacting hospital directions, regional health offices, local health districts and scientific societies. Only centers that voluntarily accessed the online platform and registered on the website were included. We have therefore no information on the characteristics of non-respondents. This may have introduced a selection bias. Nonetheless, this study describes for the first time the patterns of care provided to patients with Long-COVID at a national level. The questionnaire aimed to map the situation, provide a public list of clinical centers to patients and physicians, and define a clinical network able to explore additional research issues. All respondents were offered to participate in a national surveillance that will investigate clinical aspects with a dedicated questionnaire, based on the Global COVID-19 Clinical Platform Case Report Form for Post COVID condition (15).

In conclusion, although based on a voluntary participation and therefore subject to some possible selection bias, our survey provided information on a still minimally explored issue. The data collected indicate in the majority of centers an articulate diagnostic approach to the syndrome, multidisciplinary competences available, good integration with primary care physicians, and frequent use of differentiated technologies and multidimensional instruments. Roughly, one quarter of centers apparently provided only single or dual specialist support. These findings may inform health policies and be of help in defining common standards, interventions and guidelines that can reduce existing gaps and heterogeneity in assistance to patients with Long-COVID.

## Data availability statement

The project protocol and study data can be made available upon reasonable request. Ethics Committee consultation may be necessary in order to obtain permission to share. Requests to access the datasets should be directed to GO, graziano.onder@iss.it.

## Author contributions

MF and TG were responsible for data acquisition and directly accessed and verified the underlying data reported in the manuscript. MF performed the analyses and drafted the manuscript. Full data were available to all authors. All the authors provided substantial contribution to the study design, revised critically the manuscript, approved the final version, and agreed to be accountable for all aspect of the work.

## Funding

The survey is nested in the project Analysis and strategies of response to the long-term effects of COVID-19 infection (Long-COVID) funded by the National Center for Disease Prevention and Control of the Italian Ministry of Health in 2021 and approved by the Ethics Committee of the ISS (ref. PRE BIO CE 01.00 0015066, 2022). The study sponsor had no role in the collection, analysis, and interpretation of data; in the writing of the report; and in the decision to submit the paper for publication.

## Conflict of interest

The authors declare that the research was conducted in the absence of any commercial or financial relationships that could be construed as a potential conflict of interest.

## Publisher's note

All claims expressed in this article are solely those of the authors and do not necessarily represent those of their affiliated organizations, or those of the publisher, the editors and the reviewers. Any product that may be evaluated in this article, or claim that may be made by its manufacturer, is not guaranteed or endorsed by the publisher.
